# Human cytomegalovirus infection among febrile hematological cancer patients at the Uganda Cancer Institute

**DOI:** 10.1128/spectrum.00481-24

**Published:** 2024-09-19

**Authors:** Ocung Guido, Margaret Lubwama, Prossy Kiconco, Alfred Okeng, Irene Najjingo, Emmanuel Aboce, Rembo Phiona, Hanifah Nabbanja, Mariam Ndagire, Kamala Eva, Wekiya Enock, Jackson Orem, Moses L. Joloba, Freddie Bwanga

**Affiliations:** 1 Department of Immunology and Molecular Biology, Makerere University College of Health Sciences, Kampala, Uganda; 2 National Tuberculosis Reference Laboratory/WHO Supranational Reference Laboratory, Kampala, Uganda; 3 Department of Medical Microbiology, Makerere University College of Health Sciences, Kampala, Uganda; 4 MBN Clinical Laboratories, Kampala, Uganda; 5 Makerere University Lung Institute, College of Health Sciences, Kampala, Uganda; 6 Uganda Cancer Institute, Kampala, Uganda; Universidade Federal do Rio de, Janeiro, Rio de Janeiro, Brazil

**Keywords:** *Hematological malignancy*, human cytomegalovirus, febrile illness, cancer chemotherapy, HCMV reactivation

## Abstract

Hematological cancers, including Leukemias and Lymphomas, and their associated chemotherapy and disease-specific factors, are linked to impaired granulocyte function and numbers, increasing the risk of opportunistic infections, often presenting as fever. Human cytomegalovirus (HCMV) is one of the significant opportunistic infections in these patients, but limited data exists on its seroprevalence and active infection burden among febrile hematological cancer patients in Uganda. We conducted a cross-sectional study from June to August 2017 at the Uganda Cancer Institute (UCI). Blood samples from 161 febrile hematological cancer patients were collected. HCMV exposure was assessed using indirect enzyme-linked immunosorbent assay for IgG and IgM antibodies, and active infection was confirmed with PCR testing and gel electrophoresis. IgG positivity indicated previous exposure, while positive IgM or PCR results indicated active infection. Overall, HCMV seroprevalence based on IgG and/or IgM positivity was 106/161 (66%). IgG alone, IgM alone, and combined IgG/IgM positivity prevalence rates were 57/161 (35.4%), 22/161 (13.6%), and 27/161 (16.7%), respectively. HCMV DNA PCR was positive in 5 of the 161 (3%) samples. Among PCR-positive patients, one (20%) was positive for IgG alone, two (40%) for IgM alone, and two (40%) for both IgG and IgM. Active infection based on positive IgM and HCMV DNA PCR was found in 23 of the 161 (14.3%) patients. Two-thirds of febrile patients with hematological malignancies in Uganda had been exposed to HCMV infection, with 14.3% showing active infection. Routine testing for active HCMV infection among febrile hematological cancer patients at the UCI is essential for timely and appropriate antiviral treatment.

**IMPORTANCE:**

In this paper, we demonstrated that over two-thirds of feverish patients with blood cancers such as leukemia at the Uganda Cancer Institute are already exposed to a type of virus infection called the human cytomegalovirus (HCMV), and 14% of the patients have active disease due to this virus. This was confirmed through finding blood samples testing positive for a type of protective antibody called IgM and also upon virus DNA detection in the blood of those patients. Routine testing for this virus is not usually done in the study settings. Our findings reveal and emphasize the importance of routinely testing blood samples for active infection with this virus among the feverish patients with blood cancers in the study settings, and prompt initiation of antiviral treatment of the actively infected patients

## INTRODUCTION

Should the current trends continue, cancer will soon surpass infectious diseases as the main cause of death and disability worldwide within the next few decades (1). Based on GLOBOCAN estimates, there are 32.6 million people living with cancer (within 5 years of diagnosis), 14.1 million new cancer cases, and about 8.8 million deaths recorded worldwide as of 2015 (1–3). This burden has shifted to less-developed countries, where it accounts for approximately 57% of new cancer cases, 65% of cancer deaths, and 48% of the 5-year prevalent cancer cases reported in 2012 (4). Sub-Saharan Africa, in particular, is experiencing a marked increase in this burden, with more than 1 million incident cancers and nearly 800,000 cancer-related deaths projected annually by 2030, representing an 85% increase from 2008 (5).

Among the cancers occurring in sub-Saharan Africa, hematological malignancies have emerged as a major cause of morbidity, and account for almost 10% of cancer death in the region (2, 5, 6). These malignancies arise in hematopoietic tissue (blood-forming tissue) such as the bone marrow, or in the cells of the immune system. Examples of hematologic cancers include lymphomas (Hodgkin lymphoma and non-Hodgkin lymphoma), leukemia’s (acute myeloid leukemia, chronic myeloid leukemia, acute lymphoblastic leukemia, and chronic lymphoblastic leukemia), and myeloma (multiple myeloma) (7–9).

Hematological cancers with or without the use of chemotherapy are associated with the impairment of granulocyte number and function, predisposing such patients to viral, bacterial, fungal, and parasitic infectious complications, which often manifest as fever (10–12). Fever is often the first and only symptom of infection prompting the initiation of empirical antibacterial therapy (13–16). Additional empirical antifungal therapy is often started in cases of persistent fever (17–19). However, persistent fever lasting 4–5 days remains unexplained in 30–50% of febrile patients with no detectable evidence of clinically or microbiologically defined bacterial/fungal infection. Presence of persistent fever despite adequate antimicrobial/antifungal therapy suggests that the fever could be due to viremia and not necessarily related to the latter two infections (20, 21). With the concurrent presence of immunosuppression in patients with hematological malignancies, greater susceptibility to viral pathogens such as HCMV has emerged, which may result from the reactivation of latent infection or, rarely, from acquisition of a new infection (15, 16).

The HCMV, formally designated human herpesvirus 5 (HHV-5), is a member of the *Betaherpesvirinae* subfamily of the *Herpesviridae* family, and it is the largest member of the human herpesviruses that was first isolated from the human salivary glands (16). It contains a linear dsDNA genome with 164 non-overlapping open reading frames. According to the Centers for Disease Control and Prevention, the HCMV can stay in body fluids such as saliva, urine, blood, tears, semen, and breast milk from where it gets passed on to the new victim via direct contact with these fluids, sexual contact, breast milk to nursing infant, transplanted organs and blood transfusions. *Trans* placental spread to the fetus if the mother gets infected during pregnancy causes congenital CMV infection. The CDC further reports that in the United States, nearly one in three children is already infected with CMV by age 5, and over half of adults have been infected by age 40. Once CMV is in a person’s body, it stays there for life and can be reactivated. A person can also be re-infected with a different strain of the virus. In humans, the virus can infect various cell types including epithelial, endothelial, neuronal, smooth muscle and fibroblast cells. Primary CMV infection is usually asymptomatic or presents with subclinical/nonspecific symptoms such as fever, rash, sore throat, enlarged tonsils, and leukocytosis (16). After infection, the virus remains dormant in monocytes/macrophages in the immunocompetent host (16). However, in the immunocompromised hosts, it may reactivate and present with CMV viremia fever, CMV pneumonitis characterized by shortness of breath, cough, muscle aches, weakness or CMV retinitis presenting with blurry vision or loss of vision (16).

In the absence of effective antiviral prophylaxis, the incidence of HCMV among patients with hematological malignancy ranges from 5 to 75% (22). T-cell function is paramount in the control of HCMV, increased use of aggressive chemotherapy and T-cell-depleting agents such as alemtuzumab used to treat cancer appears to increase the risk of HCMV disease in patients with hematological malignancy following its reactivation (15). In these immunocompromised subjects, primary/reactivation infection is followed by a much more serious disease especially on those undergoing chemotherapy, where it manifest as febrile and sometimes life-threatening disseminated disease (15). As reported elsewhere, the risk of HCMV reactivation and severity of the resulting clinical manifestation ranges between 70% and 80% in patients with underlying hematological malignancy, while those receiving outpatient regimens for solid tumors have a recurrent rate that is generally less than 10–50% (16, 23).

However, among the hematological cancer patients at the Uganda Cancer Institute (UCI), the contribution of HCMV as a cause of such fever remains poorly understood. Studies conducted in Brazil, from 2008 to 2010 to assess HCMV seroprevalence in 470 patients with hematologic disorders reported an overall HCMV seroprevalence of 89%. However, the study was limited to sickle-cell anemia, hemophilia, and hemoglobinopathies (24). Another study conducted among 68 children with acute lymphoblastic leukemia in Egypt (2001–2003) using enzyme-linked immunosorbent assay (ELISA) found a seroprevalence of HCMV IgG antibody in 100% of either leukemic children or controls. However, this study was limited to children with acute lymphoblastic leukemia; it had a small sample size that was analyzed over 16 years ago and only assessed for HCMV prior exposure (25). A recent study conducted in Sudan in 2015 that aimed at determining the seroprevalence of HCMV among 70 leukemic patients using ELISA, reported an HCMV IgG seroprevalence of 75%. However, this study only focused on leukemic patients with prior exposure status (26). Since the above studies were conducted elsewhere, they cannot be used to inform the Ugandan situation due to the lack of data on HCMV. We investigated the burden of HCMV as a potential viral contributor to febrile illnesses in patients with hematological malignancies at the UCI.

## MATERIALS AND METHODS

### Study design, study site, and participants

This was a cross-sectional study conducted from June to August 2017 conducted at the UCI and at MBN Clinical Laboratories.

The UCI is an 80-bed hospital owned by the Ministry of Health Kampala Uganda in collaboration with the Fred Hutchinson Cancer Center, Seattle, Washington, USA. It serves as an East African center of excellence in cancer treatment, prevention, Research and training, serving Uganda, Kenya, Tanzania, South Sudan, the Democratic Republic of Congo, Rwanda, and Burundi. Patient recruitment was performed at UCI. MBN Clinical Laboratories, located at plot 28 Nakasero Road, Kampala, Uganda, is a well-equipped complex of medical laboratories ranging from hematology, clinical chemistry, immunoassays, culture, and sensitivity to complex molecular tests such as comprehensive PCR diagnostics, DNA sequencing, and related assays. The laboratory participates in external quality assessments and it is AABB-internationally accredited; it serves as a center of excellence in infectious diseases and molecular diagnostics for patient care and research. The immunological and polymerase chain reaction assays in this study were performed at MBN Clinical Laboratories.

We recruited adult and pediatric patients with a confirmed diagnosis of hematological malignancies with the respective diagnoses earlier confirmed based on histopathological examinations of peripheral blood smears, bone marrow smears, and bone marrow biopsies as routinely done at the UCI. The included patients who had been on cancer chemotherapy for at least four weeks but had an axillary temperature greater than 37.5°C, who voluntarily provided written informed consent to participate in the study from the parents/guardians plus or assent from children older than 8 but less than 18 years. Patients who were unconscious and unable to provide written informed consent were excluded from the study.

### Laboratory methods

The laboratory tests in this study included ELISA for HCMV IgG and IgM, and qualitative PCR for HCMV DNA on blood samples from the patients.

### Enzyme-linked immunosorbent assay for both IgG and IgM

Anti-HCMV IgG and IgM were individually measured using an Indirect-ELISA kit catalog 1201-11 for CMV IgG and 1202-2 for CMV IgM ELISA (Diagnostic Automation, Inc, Woodland Hills, California, USA) according to the manufacturer’s instructions. Briefly, diluted patient serum was added to the wells containing purified HCMV antigens coated onto the surfaces of the microwells. The HCMV IgG- or IgM-specific antibody, if present was allowed to bind to the antigen. All the unbound materials were washed away. The enzyme conjugate, which binds to the antibody-antigen complex was added. Excess enzyme conjugate was washed off and TMB Chromogenic substrate was added. The enzyme-conjugate catalytic reaction was stopped at a specific time. The intensity of the generated color was proportional to the amount of HCMV IgG- or IgM-specific antibodies in the sample. The results were then read using a microplate reader at 450 nm, and compared in parallel with the incorporated calibrator and controls. An ELISA index of 1.0 or greater was considered positive. Samples were considered negative if the ELISA index was less than 0.90. Results were considered equivocal if the ELISA index was between 0.91 and 0.99.

### Extraction of CMV DNA from whole blood

DNA was extracted from 150 µL of whole blood lysed in 100 µL of 10% sodium dodecyl sulfate and then incubated at 65°C for 10 min, followed by 100 µL of 3 N Sodium acetate. The supernatant was subsequently purified by phenol-chloroform extraction and ethanol precipitation. The dried pellet containing the DNA was then eluted in 100 µL of PCR water and stored at −20°C until use.

### PCR reagent mix

In the Pre-PCR laboratory, HCMV PCR master mix reactions were setup as indicated in Table 1.

**TABLE 1:** HCMV DNA PCR amplification reagent mix

PCR recipe	PCR reagents (stock concentration)	PCR reagents (working concentration)	PCR reaction volume (µL)	PCR reaction volume (µL) for *N* samples
PCR water (Promega)			7.0	7.0 N
PCR custom mix (Thermofisher)	10×	~1×	1.5	1.5 N
Q-solution (Qiagen)	5×	~0.5×	1.5	1.5 N
Magnesium chloride (Qiagen)	25 mM	1.4 mM	0.8	0.8 N
CMV US8 F (27)	1,000 ng/µL	107 ng/µL	1.5	1.5 N
CMV US8 R (27)	1,000 ng/µL	107 ng/µL	1.5	1.5 N
Taq polymerase (Qiagen)	5 units/µL	0.07 units/µL	0.2	0.2 N
Total			14.0	14.0 N

For the detection of HCMV DNA, PCR primers targeting the non-coding US8 region, as previously described by Soeten et al. (27), were obtained from integrated DNA Technologies (Coralville, San Diego, CA USA). The PCR primers sequences (5′-3′) were used in this study CMV US8 F **–** GGATCCGCATGGCATTCACGTATGT and CMV US8 R **–** GAATTCAGTGGATAACCTGCGGCGA.

### Amplification of HCMV DNA

To 14.0 µL of the PCR reaction mix, 10.0 µL of the eluted DNA was added and the sealed PCR tubes were transferred to the amplification laboratory where a GTQ 96 thermocycler (Hain Life Sciences, Nehren, Germany) was used to amplify the DNA template under the following parameters: Initial heating step at 95°C for 5 min, followed by 35 repeating cycles of DNA denaturation at 95°C for 30 s, primer annealing at 55°C for 20 s, and extension at 72°C for 90 s. Finally, the amplicons were held at 4°C until removed from the thermocycler.

### Gel electrophoresis detection of amplicons

**Fig 1:**
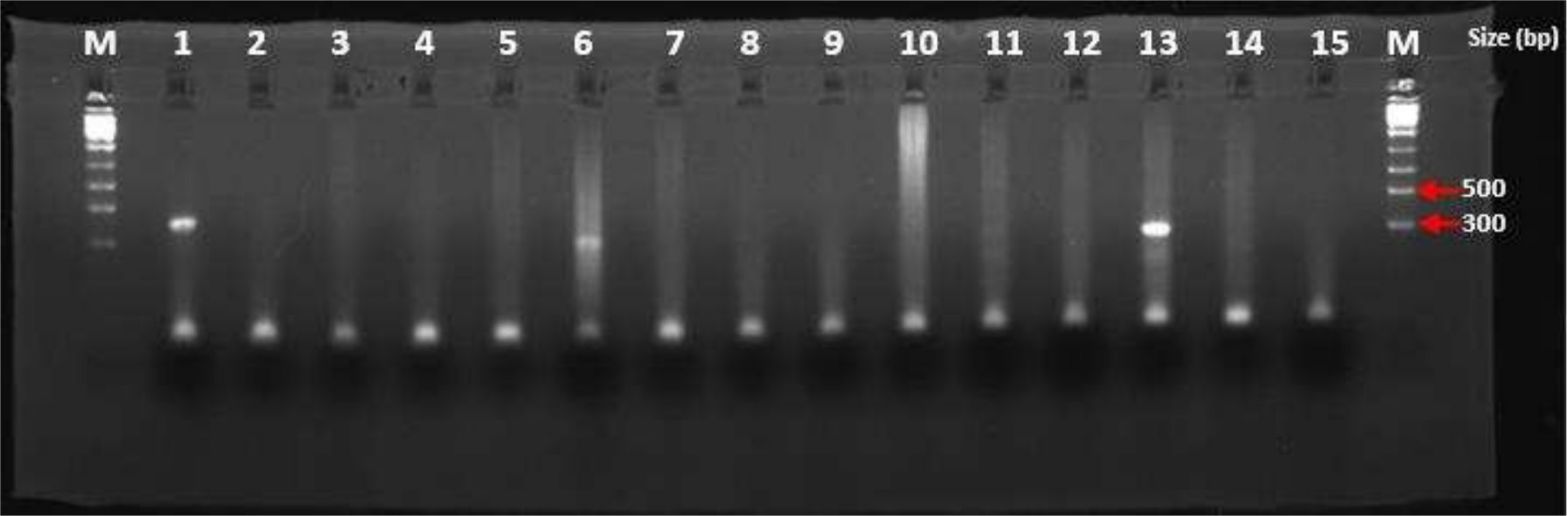
Visual representation of results from one of the agarose gel electrophoresis runs Lanes: M = 1 kb ladder (Solis Biodye), 1 = Positive control, 2 = Negative control (PCR water); lanes: 3, 4, 5, 7, 8, 9, 10, 11, 12, 14, and 15 were Negative for HCMV, lanes: 6 and 13 were positive for HCMV.

The DNA amplicons were resolved on electrophoresis using 2% agarose gel (Sigma) in 1% Sodium Borate buffer stained with 7.5 µL of 5 mg/mL ethidium bromide, run at 120 V (constant voltages, variable current) and examined under UV transilluminator and photographed. Positive (Plasmid) and negative (PCR water) controls were included for every experimental run performed. The band at the 409 bp fragment was considered positive for HCMV DNA (Fig. 1).

## RESULTS

A total of 161 participants with hematological malignancy presenting with febrile illness were evaluated for HCMV infection from June to August 2017. Of these, 86 (53%) were females. The median age was 29 years (interquartile range: 17–43). Here, 128 (80%) were on intensive chemotherapy regimen, while 33 (20%) had received the less intensive chemotherapy regimen (Table 4). Majority of the patients had acute lymphoblastic leukemia at 27%, followed by non-Hodgkins lymphoma (NHL) and acute myeloid leukemia each at 19%, as detailed in Table 2.

**TABLE 2:** Socio-demographic and clinical characteristics of the participants (*N =* 161)^*a*^

Parameter	*N* (%)
Gender	
Male	75 (47)
Female	86 (53)
Age in years	
Median (IQR)	29 (17–43)
0–17 years	42 (26)
Above 18 years	119 (74)
Chemotherapy regime	
Intensive	128 (80)
Less intensive	33 (20)
Hematological malignancy	
Acute lymphoblastic leukemia (ALL)	43 (27)
Non-Hodgkins lymphoma (NHL)	30 (19)
Acute myeloid leukemia (AML)	30 (19)
Chronic myeloid leukemia (CML)	18 (11)
Hodgkins lymphoma (HL)	18 (11)
Chronic lymphocytic leukemia (CLL)	10 (6)
Multiple myeloma (MM)	12 (7)
Education	
None	12 (8)
Primary	55 (34)
Secondary	52 (32)
Tertiary	42 (26)
No. of household occupants	
0–6	89 (55)
7 and above	72 (45)
Treatment intervention	
Antibiotic	114 (71)
Blood transfusion	113 (70)
Steroid therapy	120 (75)
HIV status	
Unknown	13 (8)
Positive	14 (9)
Negative	134 (83)

^*a*^ Key: IgG = immunoglobulin G, IgM = immunoglobulin M, IQR = interquartile range.

Of the 161 febrile patients evaluated with hematological malignancy, HCMV seroprevalence based on IgG and/or IgM antibody positivity was found to be 106/161 (66%). HCMV seroprevalence based on IgG antibody positivity was 84/161 (52%) and IgM positivity 49/161 (30%). HCMV seroprevalence based on IgG alone, IgM alone, and combined IgG/IgM antibody positivity was 57/161 (35.4%), 22/161 (13.6%), and 27/161 (16.7%), respectively.

HCMV DNA was detected in 5/161 (3%), as shown on Fig. 1. Of these, one was positive for IgG alone, the other two were positive for IgM alone, and the remaining two were seropositive for both IgG and IgM.

### Analysis for factors associated with HCMV active infection

Upon bivariate analysis, seven variables had *P* value < 0.2, as indicated in Table 3.

**TABLE 3:** Bivariate analysis using Fisher exact chi-square test^*a*^

*N* = 161	Presence of HCMV IgM Number (%)	Presence of HCMV IgG Number (%)	Chi-square	*P* value
Gender				
Male	39 (24.2%)	23 (14.3%)	1.3644	0.534
Female	45 (27.9%)	26 (16.1%)		
Age			2.1316	0.444
<17 years	14 (8.7%)	11 (6.8%)		
≥18 years	70 (43.5%)	38 (23.6%)		
Education			8.7046	0.141
None	4 (2.5%)	2 (3.7%)		
Primary	27 (16.7%)	22 (13.6%)		
Secondary	28 (17.4%)	18 (11.2%)		
Tertiary	25 (15.5%)	7 (4.3%)		
Household occupants			6.2612	0.037
0–6	47 (29.2%)	20 (12.4%)		
7 and above	37 (22.9%)	29 (18.0%)		
Chemotherapy regime			1.1469	0.734
Intensive	66 (40.9%)	38 (23.6%)		
Less intensive	18 (11.2%)	11 (6.8%)		
Hematological malignancy				
HL	12 (7.4%)	9 (5.6%)	4.8663	0.062
NHL	15 (9.3%)	6 (3.7%)	4.1296	0.097
AML	13 (8.1%)	11 (6.8%)	0.8530	0.481
CML	12 (7.5%)	8 (4.9%)	2.2117	0.421
ALL	18 (11.2%)	11 (6.8%)	2.3826	0.387
CLL	6 (3.7%)	1 (0.6%)	2.5627	0.363
MM	8 (4.9%)	3 (1.9%)	0.5692	0.821
Intervention				
Antibiotic	60 (37.3%)	35 (21.7%)	4.1742	0.154
Blood transfusion	53 (32.9%)	33 (20.5%)	0.2967	0.878
Steroid therapy	62 (38.5%)	28 (17.4%)	11.9211	0.003
HIV status			10.3114	0.104
Unknown	4 (2.5%)	5 (3.1%)		
Positive	8 (4.9%)	2 (1.2%)		
Negative	72 (44.7%)	42 (26.1%)		

^*a*^ Abbreviations: ALL = acute lymphoblastic leukemia, NHL = non-Hodgkins lymphoma, AML = acute myeloid leukemia, CML = chronic myeloid leukemia, HL = Hodgkins lymphoma, CLL = chronic lymphocytic leukemia, MM = multiple myeloma, HIV = human immunodeficiency virus.

Upon multivariate analysis, only steroid therapy had a *P* value that was less than 0.05 [odds ratio (OR): 0.36, 95% confidence interval (CI): 0.17–0.79, *P* = 0.01) as shown in Table 4.

**TABLE 4:** Multivariate logistic regression analysis of factors associated with active HCMV infection

*N* = 161	Adjusted OR	*P* value	95% CI
Education	0.83	0.35	0.56–1.23
Household occupants	1.82	0.11	0.87–3.81
Hematological malignancy			
HL	2.44	0.10	0.84–7.06
NHL	0.76	0.59	0.29–2.02
Intervention			
Antibiotic	1.17	0.71	0.51–2.67
Steroid therapy	0.36	0.01	0.17–0.79
HIV status	1.00	0.99	0.55–1.82

## DISCUSSION

In this cross-sectional study, we investigated the burden of human cytomegalovirus in patients with hematological cancers on chemotherapy presenting with febrile illness at UCI. The overall HCMV seroprevalence based on IgG and/or IgM antibody positivity was found to be 106/161 (66%). While HCMV seroprevalence based on a positive IgG alone was detected in 57/161 (35.4%), which is suggestive of prior infection with HCMV. This finding is in concordance with earlier studies from Sudan by Dafalla (2015) (28) and de Matos et al. (24) from Brazil, who reported an HCMV IgG seroprevalence of 76% among Leukemic patients and 89% in hematologic disorder patients, respectively (24, 26). After a person has been exposed to HCMV, they will have some measurable level of HCMV IgG antibody in their blood for the rest of their life. And as such HCMV IgG antibody testing should be done, alongside HCMV IgM testing, to help confirm the presence of a recent or previous HCMV infection.

In our study, a positive HCMV IgM alone was observed in 22 of the 161 (13.6%) analyzed serum samples. The positive HCMV IgM result indicates a recent infection (primary, reactivation, or reinfection). This is consistent with other studies done in Nigeria and Egypt in which seropositivity of 26% and 19% HCMV IgM were, respectively, reported among patients undergoing chemotherapy for hematological malignancy (29, 30). HCMV IgM results alone should not be used to diagnose HCMV infection. In case of suspected active HCMV infection, this may need to be confirmed through molecular detection of HCMV DNA.

In our study, HCMV DNA PCR positivity was detected in 5 of the 161 (3%) analyzed whole blood samples. Among the five, one was IgG alone positive, two were IgM alone positive, while the remaining two were both IgG and IgM seropositive. A positive IgG and DNA PCR is suggestive of reactivated latent infection, such individuals are considered more susceptible to primary infection. Positive IgM and DNA PCR indicate recent infection (primary, reactivation, or re-infection). When both HCMV IgM and IgG are positive, this may imply a seroconversion to IgM positive which is indicative of recent infection, and thus suggests that the acute illness may be associated with HCMV as confirmed by the positive DNA PCR. However, a primary infection from reactivation or re-infection cannot be definitively determined unless the patient’s previous HCMV status is known.

These findings are consistent with earlier published observations. Catalan et al. (2016) (31) reported that 16 out of 169 samples (9.5%) were positive for HCMV DNA, and Sheen et al. (32) reported 26 out of 252 samples (10.3%) as positive for HCMV DNA (31, 32). HCMV IgG and IgM results should not be used alone to diagnose HCMV infection. However, such results need to be considered in conjunction with clinical presentation, patient history, and other laboratory finding. In this context, therefore, testing for HCMV using DNA-PCR in hematologic malignancy patients presenting with febrile illness may prove a very useful technique in the rapid diagnosis of active HCMV infection thus prompting pre-emptive antiviral therapy against HCMV. This therapeutic strategy unlike prophylaxis and treatment for established HCMV disease has the advantage for preventing progression to end-organ disease, reducing exposure to antiviral toxicity, and maximizing cost–benefit ratio.

In multivariate analysis, only steroid therapy had a *P* value of less than 0.05 (adjusted OR: 0.36, 95% CI: 0.17–0.79, *P* = 0.01), suggesting that steroid use is associated with HCMV active infection among febrile patients with hematological malignancy.

Finally, although a limited number of a substantially heterogeneous patient population was studied, the lack of other identified agents coincident with fever suggests that HCMV may be the probable cause of persistent febrile illness lasting more than 4 days in this population despite adequate empiric antimicrobial therapy.

### Conclusion

Among the febrile hematological cancer patients at the UCI, the seroprevalence of HCMV based on IgG and/IgM positivity was found be 66%. Active HCMV infection based on a positive IgM and DNA PCR was detected in 14% of the patients. Steroid therapy appeared as the most relevant factor associated with active HCMV infection.

## Data Availability

The data sets used and/or analyzed during the current study are available from the corresponding author on reasonable request
